# The functional role of Pescadillo ribosomal biogenesis factor 1 in cancer

**DOI:** 10.7150/jca.58982

**Published:** 2022-01-01

**Authors:** Yong-Zhi Li, Cheng Zhang, Jun-Peng Pei, Wan-Chuan Zhang, Chun-Dong Zhang, Dong-Qiu Dai

**Affiliations:** 1 Department of Gastrointestinal Surgery, The Fourth Affiliated Hospital of China Medical University, Shenyang 110032, China.; 2 Department of Gastrointestinal Surgery, Graduate School of Medicine, The University of Tokyo, Tokyo 113-0033, Japan.; 3 Cancer Center, The Fourth Affiliated Hospital of China Medical University, Shenyang 110032, China.

**Keywords:** PES1, cancer, signaling pathway, treatment, proliferation

## Abstract

Tumors are neogrowths formed by the growth of normal cells or tissues through complex mechanisms under the influence of many factors. The occurrence and development of tumors are affected by many factors. Pescadillo ribosomal biogenesis factor 1 (PES1) has been identified as a cancer-related gene. The study of these genes may open up new avenues for early diagnosis, treatment and prognosis of tumors. As a nucleolar protein and part of the Pes1/Bop1/WDR12 (PeBoW) complex, PES1 is involved in ribosome biogenesis and DNA replication. Many studies have shown that high expression of PES1 is often closely related to the occurrence, proliferation, invasion, metastasis, prognosis and sensitivity to chemotherapeutics of various human malignant tumors through a series of molecular mechanisms and signaling pathways. The molecules that regulate the expression of PES1 include microRNA (miRNA), circular RNA (circRNA), c-Jun, bromodomain-containing protein 4 (BRD4) and nucleolar phosphoprotein B23. However, the detailed pathogenic mechanisms of PES1 overexpression in human malignancies remains unclear. This article summarizes the role of PES1 in the carcinogenesis, prognosis and treatment of multiple tumors, and introduces the molecular mechanisms and signal transduction pathways related to PES1.

## Introduction

Tumors are neogrowths formed by the growth of normal cells or tissues through complex mechanisms under the influence of many factors. The global incidence and mortality of cancer are increasing rapidly, and cancer may become the main cause of death in every country/region in the 21^st^ century 1. After tumor cells proliferate in large quantities, the malignant cells migrate from the original site to other sites through lymph, blood, implantation metastasis or direct metastasis, and then continue to grow in the new site and form the same type of tumor, thereby causing metastasis and invasion 2. However, the proliferation, metastasis and invasion of tumors are affected by many factors, which provide novel possibilities for tumor treatment.

PES1, also known as Pescadillo or NOP7, is a protein coding gene originally identified in the embryos of zebrafish which is related to DNA replication and ribosome biogenesis 3, 4. PES1 has been found to play an indispensable role in cell proliferation and may be involved in the process of oncogenic transformation and tumor progression 5. The PES1 gene encodes a nucleolar protein 5, 6 of 582 amino acids 3 whose localization is regulated by nucleolar phosphoprotein B23 7. PES1 is an important part of telomerase assembly in which PES1, telomerase reverse transcriptase (TERT) and telomerase RNA (TR) form a complex. PES1 can directly interact with TERT to control telomerase activity, maintain telomere length and regulate cell senescence 8. PES1, an essential 60S-preribosomal assembly factor, is also critical for ribosome biogenesis, nucleolar assembly and mammalian cell proliferation 9. The interaction of Pes1, block of proliferation 1 (Bop1) and WD repeat protein 12 (WDR12) controls nucleolar localization and assembly of the PeBoW complex required for maturation of the 60S ribosomal subunit 10. PES1 mRNA is widely expressed during the first three days of zebrafish embryogenesis, which indicates that it is closely related to the zebrafish embryogenic process, and is also involved in Xenopus pronephros development 3, 11.

A large number of studies have shown that PES1 is related to tumor cell proliferation, invasion and metastasis of multiple types of cancer, including prostate cancer 12, 13, liver cancer 14-18, pancreatic cancer 19, papillary thyroid cancer (PTC) 20, breast cancer 21-24, ovarian cancer 25, gastric cancer (GC) 26, neuroblastoma 27, colon cancer 28, and others. There are also some studies showing that PES1 is related to the prognosis of some cancers 14, 28.

PES1 is involved in a variety of pathophysiology. In addition to sugar metabolism and the cell replication cycle 29, highly expressed PES1 promotes tumor occurrence and progression by promoting tumor cell proliferation, invasion and migration. PES1 exerts its effects through several signaling pathways, including those of phosphatidylinositol 3'-kinase (PI3K)/AKT/glycogen synthase kinase 3β (GSK3β)/cyclin D1, β-Catenin/transcription factor (TCF), c-Jun NH2 terminal kinase (JNK), c-Myc, hypoxia inducible factor (HIF)-1α and vascular endothelial growth factor (VEGF). The purpose of this review is to summarize for the first time the different roles of PES1 in various types or subtypes of tumors and to describe the potential molecular mechanisms and their clinical significance (Tables 1 and 2).

### The Structural Characteristics of PES1

The PES1 gene is located at chromosome 22q12.2 and contains 19 exons in humans. The sequence and function of PES1 are well conserved among different species 3; human PES1 has 40% sequence homology with the Pescadillo homolog of yeast, and 79% sequence homology with the zebrafish Pescadillo homolog 5. The gene encodes a nucleolar protein which contains 588 amino acids in humans, 582 amino acids in zebrafish and 605 amino acids in yeast. The PES1 protein contains the BRCT domain 30, which can interact with the C-terminus of breast cancer associated gene 1 (BRCA1). The BRCT domain of the PES1 protein is essential for rRNA processing and nucleolar localization 31. BRCT domains have been found in several proteins related to DNA repair, cell cycle control and recombination 32-34. There are acidic amino acid clusters of glutamic acid and aspartic acid at the C-terminus of PES1 protein. PES1 protein also has many presumptive tyrosine/serine/threonine phosphorylation sites. Moreover, the PES1 protein contains a conserved site for SUMOylation 5, 21. PES1, BOP1 and WDR12 form the PEOW complex, which promotes cell proliferation by promoting maturation of 5.8S and 28S ribosomal RNAs and 60S ribosomal subunit maturation. In addition, PES1, TERT and TR form a complex. PES1 can directly interact with TERT to regulate telomerase activity, maintain telomere length and control cellular senescence. PES1 promotes telomerase assembly by inducing a direct interaction between TERT and TR 8.

### The role of PES1 in different tumors

**Figure 1:**
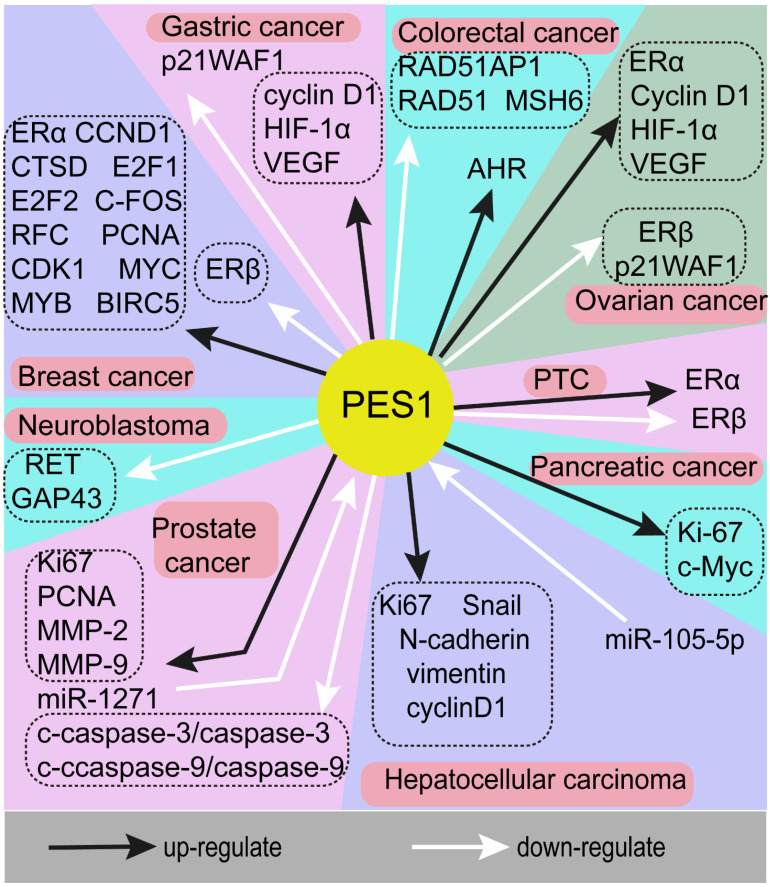
** The genes associated with PES1 in different cancers.** Black arrows indicate upregulation, white arrows indicate downregulation. As an oncogene, PES1 exerts a cancer-promoting effect through a variety of molecular mechanisms in the occurrence and development of various cancers.

A series of studies has indicated that PES1 is highly expressed in a variety of tumors and plays a crucial role in promoting cancer. This means that PES1 may be a potential key biomarker of adverse consequences and a potential molecular target in cancer treatment. However, the molecular mechanisms underlying the role of PES1 in different tumors are complex. Here, we will describe the tumors related to PES1 and the molecular mechanisms of PES1 in tumors as a starting point from which to explore potential therapeutic targets for patients with malignant tumors. The genes associated with PES1 in different cancers are summarized in Fig. 1.

### Breast cancer

PES1 is highly expressed in breast cancer cells and promotes breast tumor growth by regulating the ratio between estrogen receptor (ER)α and ERβ 23, 24. PES1 was reported to increase the transcription of luciferase reporter gene constructs containing an ER response element (ERE) in response to the ERα-specific agonist propylpyrazoltriol, but in response to the ERβ-specific agonist diarylpropionitrile, ERE reporter gene transcription was reduced. Immunohistochemical (IHC) staining results in 116 breast cancer tissues and 92 adjacent normal breast tissues showed that the expression of PES1 protein was positively correlated with the protein level of ERα and negatively correlated with the protein level of ERβ (P <0.0001). Highly expressed PES1, which was recruited to the promoter regions of ERα and ERβ, increased the homodimerization of ERα while reducing the homodimerization of ERβ and the heterodimerization of ERα-ERβ. PES1 also increased the promoter occupancy of ERα and reduced the promoter occupancy of ERβ. Thus, PES1 differentially regulates the transcriptional activity of ERα and ERβ 35.

Interestingly, after PES1 knockdown, the level of ERα and ERβ protein expression changed, but the level of ERα and ERβ mRNA did not change significantly, which indicated that PES1 regulated the expression of ERα and ERβ at the post-transcriptional level 23. By regulating the AF2 domains of ERα and ERβ, the 221-322 amino acid region of PES1 upregulated the protein level of ERα, and the 311-358 amino acid region of PES1 downregulated the expression of ERβ protein 23. After PES1 knockdown, the ubiquitination of endogenous ERα was promoted, while the ubiquitination of endogenous ERβ was inhibited. Therefore, PES1 regulates the stability of ERα and ERβ proteins through the E3 ubiquitin ligase CHIP. However, the expression of some other ER co-factors, including glutamate receptor interacting protein 1 and steroid receptor coactivator 1, regulates ERα and ERβ by simultaneously increasing the transcriptional activity of ERα and ERβ. The other two subtypes of ERβ, ERβ2 and ERβ5 36, 37, were also downregulated by PES1. MTT analysis and colony formation analysis showed that PES1 knockdown inhibited the proliferation of breast cancer cells, and anchor-independent growth ability test results showed that PES1 knockdown also inhibited the tumorigenicity of breast cancer cells 24.

Moreover, cDNA microarray analysis and RT-PCR results showed that PES1 regulates the expression of four estradiol (E2)-regulated genes, cyclin D1 (CCND1), cathepsin D (CTSD), E2F transcription factor 1 (E2F1) and c-Fos 23. After PES1 knockdown, the expression of many estrogen-responsive genes related to DNA replication and regulation of the cell cycle decreased, including proliferating cell nuclear antigen (PCNA), minichromosome maintenance genes, replication factor C, cyclin-dependent kinase (CDK) 1, E2F1, E2F2, MYC, MYB, CCND1 and surviving 38, 39. In contrast, the CDK inhibitor p27Kip1 was significantly upregulated (P<0.05). Similar to ERα and ERβ, PES1 was also recruited to the promoter regions of CTSD, CCND1, E2F1 and CCNE2. Interestingly, PES1 was recruited into the distal enhancers of ERα and ERβ, but not into the distal enhancers of CTSD and CCND1 40, 41. The results of electrophoretic mobility shift assays indicated that PES1 interacted with the promoter of the human heme oxygenase-1 42, which suggests that PES1 may regulate the transcription of genes that respond to estrogen through EREs. Furthermore, among breast cancer patients treated with tamoxifen, those with high PES1 expression tended to have a better prognosis 23.

### Prostate cancer

Through the analysis of microarray data in an online database, PES1 was identified as a differentially expressed gene which was upregulated in prostate cancer 12. PES1-silenced prostate cancer cells showed reduced EdU incorporation rates, increased apoptosis rates and reduced cell migration and invasion capabilities (all p <0.05). Through western blot analysis, it was found that PES1 silencing resulted in the downregulation of Ki67, PCNA, matrix metalloproteinase (MMP)-2 and MMP-9 expression, while the expression of c-caspase-3/caspase-3 and c-caspase-9/caspase-9 was upregulated (all p <0.05). Ki67 and PCNA are related to cell proliferation, while MMP-2 and MMP-9 are involved in migration and invasion 43, 44, and the caspase factors are related to apoptosis. In addition, PES1 silencing resulted in a decrease in PES1 mRNA expression in mice, as well as a decrease in tumor size (p <0.05). The tumor-promoting effect of PES1 in prostate cancer can be inhibited by miR-1271, because miR-1271 targeted PES1 and downregulated its expression 12.

### Hepatocellular carcinoma (HCC)

PES1 is an HCC-related protein that is differentially expressed between HCC tissues and adjacent normal tissues. Through western blotting and RT-PCR analyses, Wang et al. found that PES1 was upregulated in HCC tissues and cells and enhanced the proliferation and tumorigenesis of HCC 16, 17. Observed in the tumor cells of 134 samples, the expression of PES1 in 111 samples was at a high level (111/134, 82.8%), and the overall survival of patients with low PES1 expression was significantly longer than that of patients with high PES1 expression (p<0.05) 16. Moreover, the expression of PES1 was related to vital state (p<0.001), sex (p<0.05), race (p<0.05) and the largest tumor size (p<0.05) of patients with liver cancer 14, 16. People with high PES1 expression in liver cancer have a high risk of death. Therefore, PES1 may be a potential biological indicator for judging the prognosis of HCC. PES1 was shown to have moderate value for the diagnosis of liver cancer (AUC=0.724) 14.

PES1 promoted the proliferation, migration, invasion and tumorigenesis of liver cancer cells 14, 16-18. After knocking down PES1, the expression of Ki67 protein decreased, the number of cells was significantly reduced and the growth of liver cancer cells in mice was decreased 17. PES1 modulated the proliferation and tumorigenesis of HCC by regulating the PI3K/AKT/GSK3β/cyclin D1 signaling pathway 16. GLUT1, ENO1 and PKM2 promote glycolysis 45-47, while FBP1 and PCK1 inhibit glycolysis 48, 49. After knocking down PES1, Fan et al. found that the mRNA levels of GLUT1, ENO1 and PKM2 were downregulated, while the mRNA levels of FBP1 and PCK1were upregulated, and glucose uptake and lactate excretion of liver cancer cells were reduced, which indicated that glycolysis in HCC was reduced 17. This mechanism may affect the “Warburg effect” of HCC 50, 51. Moreover, PES1 was upregulated by BRD4 and acts as a key mediator in the anti-tumor process of bromodomain and extra-terminal motif (BET) protein inhibitors in liver cancer 17, 52. Furthermore, Wei et al. found that CD44 regulates PES1 by regulating the expression of miR-105-5p, thereby promoting the proliferation of liver cancer cells 15. Wu et al. reported that the protein levels of N-cadherin, snail, vimentin and cyclin D1 were down-regulated after PES1 knockdown 18. Cyclin D1 is a downstream gene of the β-Catenin/TCF complex, which indicates that PES1 can activate the β- catenin/TCF signaling pathway.

### Pancreatic cancer

Through the analysis of patient data identified by the GEPIA network tool 53, PES1 was found to be highly expressed in pancreatic cancer. PES1 was observed to enhance the growth of pancreatic cancer cells *in vitro* and *in vivo* 19. Western blots and IHC analysis results revealed that PES1 expression was upregulated in 35 pancreatic cancer tissues compared to 21 non-tumor pancreatic tissue samples 19. After PES1 knockdown, the results of MTS, CCK8, BrdU cell proliferation and colony formation assays showed that the proliferation ability of pancreatic cancer cells decreased, while in mice, the Ki-67 protein level and the proliferative ability of pancreatic cancer cells were reduced. Moreover, knocking down PES1 increased the sensitivity of pancreatic cancer cells to BET inhibitors, AKT inhibitors and mTOR pathway inhibitors, and decreased the mRNA and protein levels of c-Myc, which is related to the sensitivity of pancreatic cancer cells to BET inhibitors. Finally, CDK5 increased the stability of PES1 protein by phosphorylation of PES1, but had no significant effect on PES1 mRNA levels.

### Papillary thyroid cancer (PTC)

The results of IHC staining showed that the expression of PES1 and ERα protein was significantly increased in PTC, while the expression of ERβ protein was significantly reduced (P < 0.001). PES1 was found to promote the proliferation, invasion and migration of human PTC cells 20. PES1, ERα and ERβ protein levels, respectively, were related to tumor size, extrathyroidal extension, lymph node metastasis, BRAFV600E mutation and TNM stage (all p <0.05) 20. As discussed previously, knockdown of PES1 reduces the level of ERα protein and increases the level of ERβ protein. ERα promotes the proliferation, invasion and migration of PTC cells and normal thyroid cells, while ERβ has the opposite effect. Therefore, highly expressed PES1 promotes the proliferation, invasion and migration of PTC cells by adjusting the ratio of ERα/ERβ protein.

### Gastric cancer (GC)

PES1 was found to be highly expressed in GC 26, 54. IHC staining results showed that in the cancer tissues of 59 patients with GC, PES1 was highly expressed in 24 tissues, while no positive staining was detected in the corresponding normal tissues adjacent to the cancer. Silencing PES1 significantly reduced the expression of cyclin D1, HIF-1α and VEGF, increased the expression of p21WAF1, and caused cell cycle arrest in the G2 or G1 phase. Li et al. found that ablation of PES1 inhibited GC proliferation and growth both *in vitro* and *in vivo* 26.

### Neuroblastoma

Nakaguro et al. reported that the PES1 expression level increased with the growth of neuroblastomas, and that PES1 expression in patients with advanced neuroblastoma was higher than that in patients with early stage 27. The expression of PES1 was associated with poor prognosis. TUNEL assay results indicated that knockdown of PES1 inhibited the growth of neuroblastoma by inducing apoptosis. The results of quantitative PCR showed that after knocking down PES1, the expression of GAP43 and RET, two genes related to differentiation, was increased 55, but the morphology of the cells did not change significantly. Moreover, all-trans retinoic acid (ATRA)-induced differentiation may regulate PES1 expression *in vivo* and *in vitro*. The nucleolus is composed of three different sub-nucleolar compartments called the fibril center, the dense fibrillar component and granular composition, from inside to the outside of the nucleolus 56. PES1 was localized to the dense fibrillar component. The nucleolus can act as a sensor of DNA damage 57, 58, and PES1 knockdown induced the expression of DNA damage marker phosphorylated H2AX (γH2AX) and delayed DNA repair 59. PES1 and nucleolin were also found to be redistributed due to DNA damage 27.

### Ovarian cancer

Similar to PTC, PES1 has been shown to be highly expressed in ovarian cancer. Li et al. found that PES1 ablation led to a decrease in the proliferation rate of ovarian cancer cells and a delay in the G2 phase of the cell cycle 25. By differentially regulating the transcriptional activity of ERα and ERβ, PES1 increased ERα protein and reduced ERβ protein. It was found that cyclin D1 was regulated by E2 in breast cancer, where it was activated by ERα and inhibited by ERβ 60, 61. Knockdown of PES1 downregulated cyclin D1 and upregulated cyclin-dependent kinase inhibitor p21WAF1 in ovarian cancer 25. HIF-1α is the target of estrogen in ovarian cancer cells and regulates the expression of VEGF 62. The expression levels of VEGF and HIF-1α decreased after PES1 was silenced 25. The results of IHC staining of 20 normal human ovarian tissues and 54 ovarian cancer samples indicated that the expression of PES1 was positively correlated with the expression of ERα (p <0.05) and negatively correlated with the expression of ERβ (p <0.05) 25.

### Colorectal cancer

Western blot analysis showed that PES1 is highly expressed in colorectal cancer 28. It was reported that PES1 promotes the proliferation of colorectal cancer cells *in vitro* and *in vivo* 28. IHC analysis showed that in the 265 colon cancer tissues, 89 tissues showed PES1 staining, while only seven of the matched adjacent control tissues showed such staining. The expression of PES1 was significantly different between colon cancer tissues and adjacent normal tissues (P <0.001) 28. Silencing of PES1 in this study inhibited the proliferation and growth of colorectal cancer cells *in vitro* and *in vivo*.

Comparing RNA extracted from PES1-silenced cells and control cells, PES1 knockdown increased mRNA levels of RAD51, MSH6 and RAD51AP1, as shown by cDNA microarray analysis and quantitative PCR 59. RAD51 protein is essential for homologous recombination repair of DNA breaks 63. RAD51AP1 enhances the ATPase activity of RAD51 in DNA repair 64. MSH6 plays an important role in DNA mismatch repair 65. However, after PES1 ablation, the protein level of RAD51 did not change significantly, which indicates that the adaptive response to increased DNA damage may cause changes in the mRNA level of PES1 66. Immunofluorescence analysis showed that PES1 ablation reduced nuclear RAD51 levels induced by DNA damage, indicating that PES1 plays a role in the process of RAD51 entering the nucleus. Moreover, as a transcription factor responsible for inducing drug metabolizing enzymes and controlling DNA damage response 67, the aryl hydrocarbon receptor was downregulated after PES1 knockdown.

In addition, the ablation of PES1 impaired the ability of cells to repair DNA damage, reduced the rate of DNA repair and made cells more sensitive to chemotherapy drugs 59, which indicated that PES1 may be involved in regulating the sensitivity of colorectal cancer to chemotherapy. Furthermore, JNK increased the transcription of c-Jun by phosphorylating serine 63 and serine 73 in the NH2 terminal transactivation domain of c-Jun 68, and c-Jun increased the activity of the PES1 promoter. Therefore, the phosphorylation of c-Jun regulated by JNK was essential for activating the expression of PES1, and there was a positive correlation between c-Jun and PES1 (r = 0.580, P <0.0001) in human colon cancer tissue 28.

## The role of PES1 in multiple signaling pathways

### β-catenin/TCF signaling pathway

**Figure 2:**
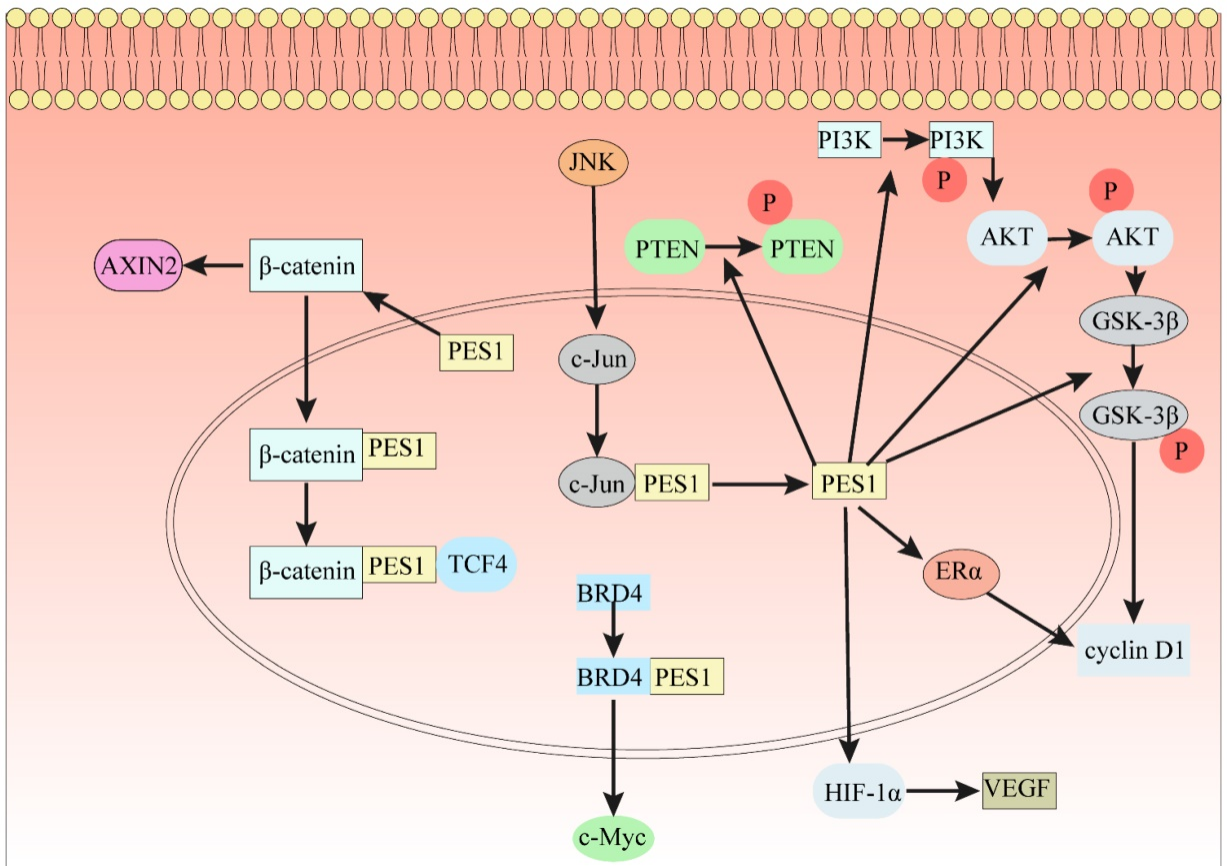
** The indispensable role of PES1 in different signaling pathways.** (1) PES1 bridges β-Catenin and TCF4 to activate the β-Catenin/TCF signaling pathway. (2) Loss of PES1 increases the level of stabilized β-Catenin in the cytoplasm and the level of protein AXIN2. (3) The JNK signaling pathway directly binds to the PES1 promoter by c-Jun to promote the expression of PES1. (4) PES1 promotes the expression of cyclin D1 by upregulating the phosphorylation levels of PTEN, PI3K, AKT and GSK3β, and also promotes the expression of cyclin D1 by upregulating ERα. (5) PES1 promotes the expression of VEGF by upregulating HIF-1α, thereby promoting angiogenesis. (6) The bromodomain of BRD4 binds to the conserved lysine sequence in PES1 and acetylates the conserved lysine sequence, which promotes the expression of c-Myc.

As is well known, β-Catenin affects gene transcription in many ways and plays an indispensable role in the occurrence and development of many cancers. In 7404 and Hep3B cells, ablation of PES1 decreased the protein expression levels of N-cadherin, Snail, vimentin and cyclin D1 18, which is a downstream gene of the β-Catenin/TCF complex. The results of GST pulldown analysis indicated that PES1 interacts with β-Catenin 18. Immunoprecipitation results in 7404 cell lysates showed that PES1 and β-Catenin formed a complex 18. PES1 bridges β-Catenin and TCF4 together and enhances their interaction, thus activating β-Catenin/TCF signaling (Fig. 2). In addition, downregulation of β-Catenin rescued the function of PES1 in promoting HCC cell migration and colony formation 18.

### Wnt/β-Catenin signaling pathway

The Wnt/β-Catenin signaling pathway regulates cell proliferation and apoptosis by inducing the expression of Wnt target genes 69, which indicates that the Wnt/β-Catenin signaling pathway plays an important role in the growth of cancer and stem cells 70. After knocking down PES1, the level of stabilized β-Catenin increased, and the expression of AXIN2 increased significantly 71. Knockdown of PES1 triggered nucleolar stress and further activated the Wnt/β-Catenin pathway 71. This indicates that the loss of the key nucleolar factor PES1 can trigger nucleolar stress, further activate the Wnt/β-Catenin signaling pathway, and ultimately increase the expression of Wnt target protein AXIN2 (Fig. 2).

Interestingly, the overexpression of PES1 activates the β-Catenin/TCF signaling pathway, while the Wnt/β-Catenin signaling pathway is activated after PES1 is knocked down. Whether PES1 is involved in a negative feedback regulatory mechanism between the two pathways remains to be further explored.

### PI3K/AKT/GSK3β/cyclin D1 signaling pathway

Changes in the PI3K-Akt signaling pathway have been reported in many human cancers 72. The PI3K-Akt signaling pathway is an intracellular signaling pathway crucial in regulating the cell cycle. GSK-3β can phosphorylate cyclin D1 73. After knockdown of PES1 in HCC cells, phosphorylated phosphatase and tensin homolog (p-PTEN), p-PI3K, p-AKT, p-GSK-3β and cyclin D1 were all downregulated 16. After knocking down PES1 in AGS and N87 cells, in addition to a significant decrease in the mRNA level of cyclin D1, it was also found that p21WAF1, one of the cyclin-dependent kinase inhibitors, increased significantly at the mRNA level 26. Cyclin D1 is regulated by E2 in breast cancer, where it is activated by ERα and inhibited by ERβ 60, 61. Highly expressed PES1 upregulates ERα and downregulates ERβ, so PES1 may activate cyclin D1 by upregulating ERα (Fig. 2). However, the specific molecular regulatory mechanism underlying the PES1 and PI3K/AKT signaling pathway needs to be further explored.

### HIF-α Pathway

The HIF-1α pathway plays an important role in tumor metastasis and angiogenesis 74. It was reported that inhibiting HIF-1 activity had a significant effect on tumor growth 75, 76. In human squamous cell carcinoma, HIF-1α induced lymphangiogenesis and promoted tumor cell invasion by upregulating VEGF-C 77. Li et al. found that after knocking down PES1 in AGS and N87 cells, the mRNA and protein levels of HIF-1α and VEGF decreased significantly 26. The expression of VEGF was dependent on HIF-1α, so that when PES1 was knocked down, re-expression of PES1 increased the protein levels of HIF-1α and VEGF. In a word, PES1 can promote tumor invasion and lymphangiogenesis by regulating the expression of HIF-1α (Fig. 2).

### C-Myc Pathway

Numerous reports have indicated that c-Myc regulates genes associated with the biogenesis of ribosomes and mitochondria and the metabolism of glucose and glutamine 78, 79. Moreover, c-Myc induces genes involved in nucleotide metabolism and DNA replication 78. Furthermore, c-Myc plays a crucial role in cancer cell energy metabolism and promotes cancer cell proliferation. Jin et al. found that the protein level of PES1 was positively correlated with the expression level of c-Myc 19. The bromodomain-containing protein BRD4 can regulate c-Myc. Fan et al. found that knocking out BRD4 reduced PES1 expression in HepG2 and Huh7 cells 17. The bromodomains (BD1 and BD2) of BRD4 can recognize and acetylate the conserved lysine sequence in PES1. PES1 binds to BRD4 exogenously in 293 T cells and binds to BRD4 endogenously in PANC-1 cells 19. The interaction of PES1 and BRD4 is regulated by acetylation, and the deacetylation of PES1 leads to reduced binding to BRD4. Overexpression of PES1 upregulated the expression of c-Myc, but after overexpression of PES1 and knockdown of BRD4, c-Myc was not significantly upregulated 19. Compared with PANS-1 cells in which PES1 or BRD4 was knocked down alone, the expression of c-Myc in PANS-1 cells in which both PES1 and BRD4 were knocked down did not change significantly 19. In conclusion, PES1 interacts with BRD4 and promotes the expression of c-Myc (Fig. 2).

### JNK signaling pathway

The JNK signal transduction pathway is an important branch of the MAPK pathway, which plays a crucial role in a variety of physiological and pathological processes such as cell cycle, reproduction, apoptosis and cell stress. JNK signaling promotes cancer cell survival 80, 81. JNK increased the transcription of c-Jun by phosphorylating serine 63 and serine 73 in the NH2 terminal transactivation domain of c-Jun 68. Chromatin immunoprecipitation analysis results indicated that c-Jun directly binds to the promoter of PES1 and interacts with it to regulate PES1 promoter activity 28 and increase expression of PES1. In short, the JNK signaling pathway increased the expression level of PES1 by regulating the activity of the PES1 promoter (Fig. 2).

Here, we hypothesize that with PES1 as an intermediate medium, the JNK signaling pathway is likely to form a closed feedback regulatory loop with the previously introduced β-Catenin/TCF and Wnt/β-Catenin signaling pathways. Whether this hypothesis is valid or not will require more research.

### MicroRNAs, circRNA and PES1

MicroRNA (miRNA) is closely related to the occurrence, development and treatment of cancer 82. Jiang et al. found that miR-1271 can specifically bind to PES1, which was regulated as a target gene of miR-1271 12. Wei et al. found that CD44 regulates the expression of miR-105-5p, and expression of miR-105-5p further targets and regulates PES1, thereby regulating the proliferation of liver cancer cells 15. As a molecular inhibitor of PES1, circANRIL binds to the C-terminal domain of PES1, negatively regulates PeBoW activity and inhibits rRNA maturation 83.

In general, PES1 may also participate in many other signaling pathways which interact or share crosstalk with β-Catenin, PI3K/AKT, HIF-α and c-Myc, thus jointly regulating the pathways involved in the occurrence and development of tumors (Table 1).

## Conclusion and perspectives

Tumorigenesis and proliferation of tumors is a very complex process, involving the formation of blood vessels and the replication of DNA. This process not only depends on changes in genetics, but also changes in epigenetics. A large number of studies have shown that PES1 is highly expressed in a variety of human cancers. PES1 is a nucleolar protein which is involved in the biogenesis of ribosomes and regulates DNA replication. PES1 can also regulate the glycolysis of tumor cells, which suggests that PES1 may be related to the “Warburg effect”, a common feature of solid tumors. PES1 is an important part of telomerase assembly and can regulate telomerase activity, maintain telomere length and regulate cellular senescence 8. In the process of tumor cell development, the activity of telomerase is often higher, which indicates that PES1 may also regulate tumor progression by regulating the activity of telomerase. PES1 participates in the formation of the PeBoW complex and regulates the maturation of the 60S ribosomal subunit 10.

From its initial identification in zebrafish, PES1 has been studied for more than 20 years, and research on its function and related signal transduction pathways still provides surprising findings. PES1 is believed to be involved in a variety of physiological and pathological processes, including ribosome biogenesis, DNA replication and cancer 3-5. PES1 is highly expressed in many tumors and promotes the progression of cancer. PES1 may also be a potential prognostic marker of cancer. Furthermore, given the emergence of tumor resistance to drugs including BET inhibitors, PES1 may serve as a potential target to increase tumor sensitivity to drugs.

PES1 is closely related to a variety of signaling pathways. PES1 is widely involved in regulating multiple locations and branches of the Wnt signaling pathway, which suggests an inseparable relationship between PES1 and Wnt. PES1 regulates different target proteins downstream in the Wnt signaling pathway, while playing different roles in tumor development. Knockdown of PES1 may activate the Wnt/β-Catenin signaling pathway, while the JNK signaling pathway can promote the expression of PES1, which implies that PES1 may form a regulatory loop in the Wnt signaling pathway. PES1 promotes the proliferation of breast cancer, ovarian cancer and PTC by differentially regulating ERα and ERβ. PES1 regulates the glycolysis of tumor cells by changing the gene expression of GLUT1, PKM2, ENO1, FBP1 and PCK1, which provides a new approach for the treatment of cancer. If a drug can target PES1 to inhibit the glycolysis of tumor cells, it may reduce the productivity of tumor cells and inhibit their growth. In addition, PES1 promotes tumor progression and angiogenesis by increasing factors such as cyclin D1, HIF-1α and VEGF. Therefore, drugs targeting PES1 may affect tumor progression through telomerase, glycolysis, angiogenesis, and other cellular functions.

Recent studies have also found that some proteins, miRNAs and circRNAs can target the expression of PES1. These data indicate that we can regulate PES1 at the epigenetic level to achieve tumor suppression. Various data indicate that PES1 participates in a very complex intracellular molecular regulation network, and this complex network is inextricable from the occurrence and development of tumors. Therefore, further research on PES1 will help to better evaluate cancer prognosis, develop new tumor-targeted therapeutic drugs and increase tumor sensitivity to drugs.

## Figures and Tables

**Table 1:** PES1 regulates a variety of signaling pathways to promote tumor occurrence and development

Cancer	Related pathways	Role	Function	Refs
Breast cancer	-	Oncogene	Proliferation, prognosis	22, 23
Prostate cancer	-	Oncogene	Proliferation	12
Hepatocellular carcinoma (HCC)	β-Catenin /TCF; PI3K/AKT/GSK3β/cyclin D1	Oncogene	Proliferation, tumorigenesis, glycolysis	16-18
Pancreatic cancer	c-Myc	Oncogene	Proliferation, resistance to chemotherapy drugs	19
Papillary thyroid cancer (PTC)	-	Oncogene	Proliferation, invasion, migration	20
Gastric cancer (GC)	HIF-1α	Oncogene	Proliferation	26
Neuroblastoma	-	Oncogene	Proliferation, prognosis	27
Ovarian cancer	HIF-1α	Oncogene	Proliferation	25
Colorectal cancer	JNK	Oncogene	Proliferation	28

The minus sign indicates the related signaling pathway has not been discovered.

**Table 2:** Clinical significance of PES1 in various cancers

Cancer	Clinical significance	Refs
Breast cancer	Sensitivity to tamoxifen	23
Prostate cancer	-	-
Hepatocellular carcinoma (HCC)	Histologic grade, overall survival, relapse-free survival, tumor size, T classification	14, 16
Pancreatic cancer	Tumor size, sensitivity to BET inhibitors	19
Papillary thyroid cancer (PTC)	TNM stage, tumor size, extrathyroidal extension, lymph node metastasis	20
Gastric cancer (GC)	Tumor size	26
Neuroblastoma	International Neuroblastoma Staging System (INSS) stage, overall survival, relapse-free survival	27
Ovarian cancer	-	-
Colorectal cancer	Tumor size, sensitivity to anticancer drugs	28, 59

## Abbreviations

PES1: Pescadillo ribosomal biogenesis factor 1
BOP1: block of proliferation 1
WDR12: WD repeat protein 12
miRNA: microRNA
circRNA: circular RNA
BRD4: bromodomain-containing protein 4
TERT: telomerase reverse transcriptase
TR: telomerase RNA
PTC: papillary thyroid cancer
GC: gastric cancer
PI3K: phosphatidylinositol 3'-kinase
GSK3β: glycogen synthase kinase 3β
TCF: transcription factor
JNK: c-Jun NH2 terminal kinase
HIF: hypoxia inducible factor
VEGF: vascular endothelial growth factor
BRCA1: breast cancer associated gene 1
ER: estrogen receptor
ERE: ER response element
IHC: Immunohistochemical
CCND1: cyclin D1
CTSD: cathepsin D
E2F1: E2F transcription factor 1
PCNA: proliferating cell nuclear antigen
CDK: cyclin-dependent kinase
MMP: matrix metalloproteinase
BET: bromodomain and extra-terminal motif
ATRA: all-trans retinoic acid
γH2AX: phosphorylated H2AX
p-PTEN: phosphorylated phosphatase and tensin homolog
